# What’s ploidy got to do with it? Understanding the evolutionary ecology of macroalgal invasions necessitates incorporating life cycle complexity

**DOI:** 10.1111/eva.12843

**Published:** 2019-08-05

**Authors:** Stacy A. Krueger‐Hadfield

**Affiliations:** ^1^ Department of Biology University of Alabama at Birmingham Birmingham Alabama

**Keywords:** asexual reproduction, Baker's law, clonality, invasion, life cycle, macroalgae, marine, selfing

## Abstract

Biological invasions represent grave threats to terrestrial, aquatic, and marine ecosystems, but our understanding of the role of evolution during invasions remains rudimentary. In marine environments, macroalgae account for a large percentage of invaders, but their complicated life cycles render it difficult to move methodologies and predictions wholesale from species with a single, free‐living ploidy stage, such as plants or animals. In haplodiplontic macroalgae, meiosis and fertilization are spatiotemporally separated by long‐lived, multicellular haploid and diploid stages, and gametes are produced by mitosis, not meiosis. As a consequence, there are unique eco‐evolutionary constraints that are not typically considered in invasions. First, selfing can occur in both monoicious (i.e., hermaphroditic) and dioicious (i.e., separate sexes) haplodiplontic macroalgae. In the former, fertilization between gametes produced by the same haploid thallus results in instantaneous, genome‐wide homozygosity. In the latter, cross‐fertilization between separate male and female haploids that share the same diploid parent is analogous to selfing in plants or animals. Separate sexes, therefore, cannot be used as a proxy for outcrossing. Second, selfing likely facilitates invasions (i.e., Baker's law) and the long‐lived haploid stage may enable purging of deleterious mutations, further contributing to invasion success. Third, asexual reproduction will result in the dominance of one ploidy and/or sex and the loss of the other(s). Whether or not sexual reproduction can be recovered depends on which stage is maintained. Finally, fourth, haplodiplontic life cycles are predicted to be maintained through niche differentiation in the haploid and diploid stages. Empirical tests are rare, but fundamental to our understanding of macroalgal invasion dynamics. By highlighting these four phenomena, we can build a framework with which to empirically and theoretically address important gaps in the literature on marine evolutionary ecology, of which biological invasions can serve as unnatural laboratories.

## 1 INTRODUCTION

Biological invasions represent one of the gravest threats to biodiversity by altering ecosystem functioning and homogenizing native biota (Vitousek, Mooney, Lubchenco, & Melilo, 1997). Therefore, elucidating the mechanisms that facilitate invasions is a major goal of invasion biology (Kolar & Lodge, 2001), but requires an understanding of both the ecological (i.e., distributional and phenological shifts) and evolutionary strategies (i.e., adaptation and gene flow) that enable the spread and persistence of not only colonizing species, but also native species in the recipient habitats (Rey et al., 2012). Indeed, invasions can serve as model systems with which to empirically address these fundamental eco‐evolutionary questions at spatiotemporal scales that would be difficult to replicate in the laboratory or the field (Rice & Sax, 2005).

Invasion success, measured by establishment and spread in novel habitats, is likely driven by a suite of species traits and characteristics (e.g., Kolar & Lodge, 2001). Phenotypic plasticity, the property of a genotype to express different phenotypes in different environments (e.g., Bradshaw, 1965; Pigliucci, 2005; Schlichting, 1986), is thought to play an important role in invasion success (Richards, Bossdorf, Muth, Gurevitch, & Pigliucci, 2006). Invaders may be more plastic (i.e., general purpose genotypes, Baker, 1965) or there may be genetic variation in plasticity in which some genotypes with more plasticity will have an advantage in novel environments contributing to the evolution of plasticity (Bradshaw, 1965; Richards, Pennings, & Donovan, 2005). Richards et al. (2006) suggested successful invaders benefit from plasticity as either “jack‐of‐all‐trades” (i.e., robustness: invader can maintain fitness across a variety of environmental conditions), “master‐of‐some” (i.e., opportunistic: invader can take advantage of certain environmental conditions), or “jack‐and‐master” (i.e., combines both robust and opportunistic attributes). An invader that can maintain positive population growth by exploiting one of these strategies would have greater potential of successful colonization and subsequent range expansion (Hulme, 2008).

Successful colonization will also be strongly influenced by propagule pressure in terms of the sizes, numbers, and spatiotemporal patterns of arrival (reviewed in Simberloff, 2009). The production, dispersal, and genetic constitution of propagules are, in turn, largely governed by the mating system (Barrett, Colautti, & Eckert, 2008; Eckert et al., 2010; Lane, Forrest, & Willis, 2011; Pannell, 2015; Pannell et al., 2015). The mating system or reproductive mode will affect the amount of *genetic diversity within* populations and the amount of *genetic differentiation among* populations (Hamrick & Godt, 1996). In general, sexual reproduction and, more specifically, outcrossing will be associated with larger, more genetically diverse populations with higher potential for adaptation. By contrast, inbreeding will result in smaller effective population sizes, lower genetic diversity, and reduced effective recombination. Similarly, asexual reproduction will reduce the effective population size and increase the effects of genetic drift. However, heterozygosity is predicted to accumulate over time in asexual lineages (Balloux, Lehmann, & de Meeûs, 2003; Halkett, Simon, & Balloux, 2005), such that genetic diversity (as measured by observed and expected heterozygosity) could be comparable or exceed that of sexual populations (Halkett et al., 2005; see also Guillemin et al., 2008; Krueger‐Hadfield et al., 2016).

The life‐history traits that affect mating systems are evolutionarily labile and vary within and between taxa (Barrett, 2002; Bierzychudek, 1985; Kolar & Lodge, 2001; Lynch, 1984; van Kleunen, Dawson, & Maurel, 2015). In animals, studies tend to focus on the number of mates females and males may obtain (Shuster, 2009). In contrast, in angiosperms, the focus of studies has centered on the degree to which sexual reproduction involves selfing (self‐fertilization) versus outcrossing (mating among unrelated individuals) because many species are hermaphroditic and self‐compatible (Eckert et al., 2010). The axis of variation from selfing to outcrossing is complemented by an analogous axis of asexual to sexual reproduction. Sexual and asexual reproduction usually occur simultaneously in plants (Vallejo‐Marín, Dorken, & Barrett, 2010), unlike in animals where environmental cues cause switches to sexual reproduction (e.g., in cladocerans, Bell, 1992). Mating systems in other non‐animal and angiosperm taxa have been less well studied, and, as a consequence, we know much less about the relative frequencies of sexual versus asexual reproduction or selfing/inbreeding versus outcrossing (but see Billiard, López‐Villavicencio, Hood, & Giraud, 2012; Crawford, Jesson, & Garnock‐Jones, 2009; de Groot, Verduyn, Wubs, Erkens, & During, 2012; Engel, Destombe, & Valero, 2004; Engel, Wattier, Destombe, & Valero, 1999; Guillemin et al., 2008; Krueger‐Hadfield et al., 2016; Krueger‐Hadfield, Roze, Correa, Destombe, & Valero, 2015; Krueger‐Hadfield, Roze, Mauger, & Valero, 2013; Taylor, Eppley, & Jesson, 2007).

There is a clear need to quantify how human disturbance alters the selective forces that impinge on the mating system. Understanding life history traits linked to the mating system is more relevant due to range shifts, of which biological invasions are examples. Baker (1955) formalized the argument that shifts in the reproductive system should greatly facilitate colonization success (Cheptou, 2012; Pannell et al., 2015). The number of mates in a new habitat is low or even zero. Individuals or species with an enhanced capacity for uniparental reproduction (i.e., selfing, asexuality, or a combination of the two) should have an increased likelihood of successful establishment (Baker, 1955; Pannell, 2015; Pannell et al., 2015). Correlations between life‐history traits and mating systems have been found across eukaryotic taxa, including higher rates of uniparental reproduction following long‐distance dispersal (e.g., Hardy et al., 2004; Kalisz, Vogler, & Hanley, 2004).

Though uniparental reproduction may increase the chance of establishment, it may reduce resilience to rapidly changing environments as a result of lower adaptive potential due to inbreeding depression or low genotypic diversity. Indeed, the initial transition to selfing will result in high levels of inbreeding depression, and inbreeding depression is thought to play a critical role in preventing the evolution of self‐fertilization (Ågren, Oakley, McKay, Lovell, & Schemske, 2013; Charlesworth & Charlesworth, 1987; Sletvold, Mousset, Hagenblad, Hansson, & Ågren, 2013). Once deleterious mutations are purged from the population by selection, selfing can become adaptive. Pujol, Zhou, Vilas, and Pannell (2009) found low inbreeding depression at the range edges of a common European plant, easing the conditions for selfing to evolve. A history of range expansions may reverse the direction of selection on the mating system, facilitating transitions to selfing, and uniparental reproduction may occur more often in species that have recently expanded (Pujol et al., 2009), including invasive species. Similar expectations can be expected of asexual reproduction and colonization events (Barbuti et al., 2012; Cascante‐Marín et al., 2006; Cronberg, 2002; Mergeay, Verschuren, & De Meester, 2006; Patiño et al., 2013), though enhanced asexuality may limit the ability of newly established populations to track environmental change (but see as examples Orr, 2000; Verhoeven, Jansen, Van Dijk, & Biere, 2009). Thus, rates of uniparental (i.e., selfing and asexuality) versus biparental reproduction (i.e., outcrossing) are of critical evolutionary importance under invasion scenarios, as they will impact potential migration, and then subsequent adaptation, plasticity, and evolutionary potential (Pannell, 2015).

## 2 MARINE INVASIONS AND MACROALGAE

*Yet we may venture to say, that those who indulge in more than a superficial and momentary observation of them are far from numerous, and it would be scarcely truthful to speak of seaweeds as “familiar” things.* The Seaweed Collector by Shirley Hibberd (1872)

In comparison with terrestrial environments, the history, diversity, and consequences of marine invasions are poorly known for most of the world's coastlines (Bax et al., 2001), despite the magnitude of ecological and evolutionary changes caused by invaders in these ecosystems (Carlton & Geller, 1993). Many taxa that lack commercial value or lack diagnostic morphological features regularly go unrecognized (Bax et al., 2001; including marine macroalgae: Krueger‐Hadfield, Magill, et al., 2017b; Krueger‐Hadfield, Stephens, Ryan, & Heiser, 2018), or are assumed to be recent arrivals. Many “recently discovered” marine invaders are often thought to be introduced as a result of ballast water due to the timing of their “discovery,” whether or not ballast water is the appropriate vector based on the natural history of the organism (e.g., Krueger‐Hadfield, Kollars, et al., 2017a; see also Williams & Smith, 2007).

Moreover, we know less about evolution during marine invasions in estuarine and marine systems (Grosholz, 2002). The most notable exceptions include the work by Lee and collaborators in a marine copepod (Lee, 2002; Lee & Gelembiuk, 2008), as well as a recent study in an invasive macroalga (Sotka et al., 2018), in which genetic adaptation and rapid evolution were documented. It is perhaps, then, not surprising that few studies have focused on the evolutionary consequences of mating system variation in the sea. Nevertheless, mating systems will influence the extent of postintroduction adaptations, and their underlying mechanisms, but we lack evidence in the marine environment of these evolutionary events (Viard, David, & Darling, 2016). Coupled with the knowledge that invasions alter plant colonization ability (Barrett et al., 2008), and evidence of associations between dispersal and mating system in the sea (Addison & Hart, 2005; Valero, Engel, Billot, Kloareg, & Destombe, 2001), there is a critical need to explore mating system variation and evolution during invasions.

This knowledge gap is particularly relevant for green, red, and brown macroalgae in which the ecological and evolutionary consequences of their invasions are largely unknown except for a few emblematic species (Williams & Smith, 2007). Moreover, macroalgal population genetics, including descriptions of the mating system, have been undertaken in far fewer species as compared to animals or plants in all other environments (Andreakis, Kooistra, & Procaccini, 2007; Krueger‐Hadfield & Hoban, 2016; Valero et al., 2001), thereby limiting the repository of critical background information from which predictions can be made. Importantly, macroalgae also exhibit a tremendous amount of life cycle diversity, thereby complicating traditional population genetics, rendering the applicability of general rules about the eco‐evolutionary outcomes of invasions questionable, and necessitating detailed natural history data that are often lacking (Krueger‐Hadfield & Hoban, 2016).

Nevertheless, macroalgae are excellent eco‐evolutionary models with which to fill in the substantial gaps in our understanding of the responses of marine populations to climate change, particularly in the way life cycle and mating system variation intersect. They are critical ecosystem engineers in inter‐ and subtidal ecosystems worldwide (Lüning, 1990), and form the basis of lucrative aquaculture valued at $11 billion USD (FAO, 2018). What we collectively refer to as “macroalgae” are three diverse, eukaryotic lineages (Coelho, Simon, Ahmed, Cock, & Partensky, 2013), and include many invasive foundation species that have impacted near‐shore marine ecosystems worldwide (Williams & Smith, 2007). Despite their ecological, evolutionary, and applied importance, macroalgae have not received the same empirical attention as other eukaryotic lineages (Collen et al., 2014); yet, they face the same consequences of rapid environmental change and cannot simply move to a less marginal habitat as they are sessile (e.g., Vergés et al., 2014; Wernberg, Bennett, Babcock, Bettignies, & Cure, 2016).

Unlike animals and seed plants, the majority of macroalgae have complex life cycles with more than one free‐living stage (i.e., more than one free‐living individual in the same life cycle), usually differing in ploidy level (i.e., diploid and haploid), and comparable to the variation found across all other eukaryotes (Bell, 1994). As a consequence, predictions from plants and animals cannot be generally applied to algae (Krueger‐Hadfield & Hoban, 2016). Macroalgal populations exhibit life history traits suggesting within and among population levels of mating system variation that is comparable to angiosperms (Entwisle, Vis, & McPherson, 2004; Valero et al., 2001). This variation also suggests that some macroalgal populations may be more likely to be source populations of invasions (van Kleunen, Weber, & Fischer, 2010), but this has rarely been tested (but see as examples Guzinski, Ballenghien, Daguin‐Thiébaut, Leveque, & Viard, 2018; Krueger‐Hadfield et al., 2016; Krueger‐Hadfield, Kollars, et al., 2017a, Le Cam et al., 2019). Therefore, our knowledge about how climate change might affect adaptive potential in seaweeds, especially in extreme (i.e., range edges) and newly invaded environments, remains rudimentary, despite some macroalgal invaders dramatically altering the habitats into which they are introduced (Andreakis & Schaffelke, 2012; Williams & Smith, 2007).

In this perspective, I outline macroalgae as eco‐evolutionary models, with particular relevance to understanding of the role of evolution in shaping marine invasions. I highlight the subtle, but critical, differences in their life cycles that likely lead to unique eco‐evolutionary outcomes. First, I discuss life cycle diversity with an emphasis on macroalgal life cycle variation. Then, in the four subsequent sections, I highlight unique eco‐evolutionary characteristics of haplodiplontic macroalgae: (a) Selfing can occur in both monoicious (both sexes in the same individual) and dioicious taxa (separate sexes) with potential impacts on invasion dynamics; (b) while selfing is linked to inbreeding depression, the long‐lived haploid stage may allow purging of the genetic load, reducing the costs associated with selfing; (c) due to the spatiotemporal separation of meiosis and fertilization, asexual reproduction (fragmentation or propagule production) will result in the loss, potentially irrevocably, of one the free‐living ploidies and/or sexes; and, finally, (d) haploid and diploid stages may occupy different ecological niches, thereby strongly influencing invasion dynamics and mating system variation. I conclude with a framework the theoretical and empirical work necessary to understand macroalgal evolution in the face of climate change, of which invasions are an acute example, and several potential macroalgal models with which to explore these processes.

## 3 LIFE CYCLE DIVERSITY

*Why are there organisms that maintain both a haploid and a diploid phase to their life cycles in the face of variants that would allow dominance of one or the other phase?* S.P. Otto (1994) *Lectures in Mathematics in the Life Sciences* Volume 25, p. 71

Eukaryotic life cycles involve a vegetative process of growth and reproduction and a sexual process of meiosis and fertilization (Bell, 1994). Although reproduction is often linked with sexual reproduction, this is not always the case. Asexual reproduction, through spore production or vegetative propagation through fission or fragmentation, can exist instead of or in addition to the sexual cycle. Moreover, though the cyclic alternation between meiosis (reduction from diploid to haploid) and fertilization (reconstitution of diploidy) is a common feature of eukaryotic sex, there is profound variation in (a) the timing of meiosis and fertilization, (b) the proportion of time spent in the haploid and diploid phases, and (c) the degree of somatic development in each life cycle phase.

**Figure 1:**
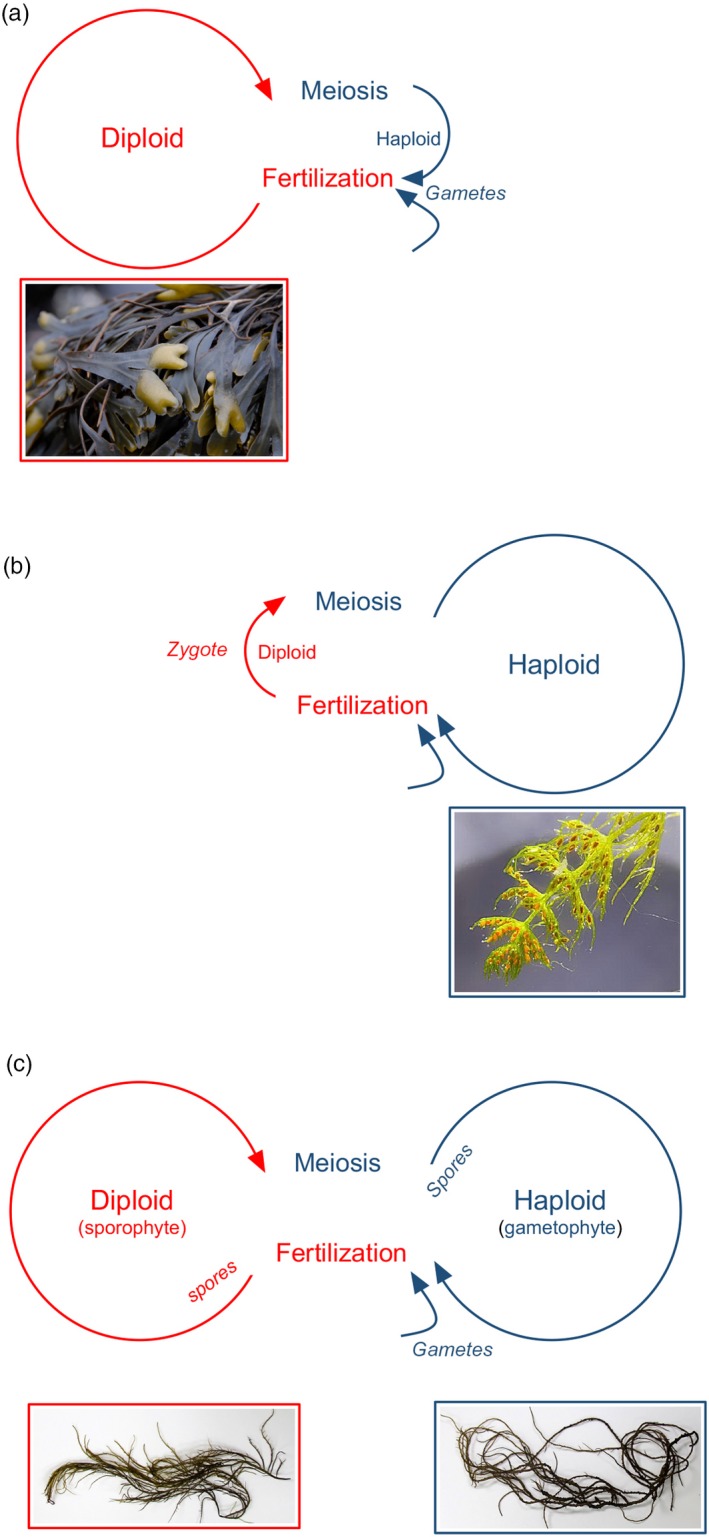
(a) Diplontic life cycles are found in animals, and in macroalgae, in the fucoids as an example shown in photograph. Somatic development occurs in the diploid stage, and the haploid stage is unicellular. (b) In haplontic life cycles, such as found in the Charophytes, shown in photograph, somatic development occurs in the haploid stage. (c) In the haplodiplontic life cycles of many macroalgae, meiosis and fertilization are spatiotemporally separated by long‐lived haploid gametophytes and diploid sporophytes, shown is the red alga *Agarophyton vermiculophyllum*. While angiosperms have haplodiplontic life cycles, the gametophyte stage is highly reduced corresponding to a pollen grain of only two or three cells and an embryo sac consisting of seven cells. (Photo credit: S.A. Krueger‐Hadfield)

There are three simplified types of eukaryotic life cycles (see, for example, Bell, 1994, as there are many unique and interesting exceptions): diplontic, haplontic, and haplodiplontic (Figure 1), and these are found across the three macroalgal lineages. If fertilization directly follows meiosis, somatic development will occur only in the diploid stage. The unicellular gametes are the haploid stage in which no somatic development occurs (Figure 1a). This life cycle is found in animals and in the macroalgae, in the Fucales, the Ascoceirales, and the Bryopsidales (Graham & Wilcox, 2000). In many animals, sex is obligately associated with reproduction, but in many diplontic algal species this is not the case. In *Caulerpa taxifolia*, for example, there is a clear separation between sexual and vegetative processes in the life cycle, despite the single, free‐living stage being diploid, and has contributed to the spread of this species outside its native range (Arnaud‐Haond, Candeias, Serrão, & Teixeira, 2013; Meusnier, Valero, Olsen, & Stam, 2004). Similarly, in the Baltic Sea, there are asexual *Fucus* populations in which clonality is an important reproductive mode in brackish waters (Tatarenkov et al., 2005).

If meiosis directly follows fertilization, by contrast, somatic development will occur in the haploid stage. The diploid stage is the zygote, and though the zygote may be a resting cyst, there is no somatic development (Figure 1b). In haplontic life cycles, gametes are produced by mitosis and are genetically identical to parental thallus. As in diplontic life cycles, sex is not obligately linked with reproduction. The invasive charophyte *Nitellopsis obtusa* is thought to be spreading throughout the Great Lakes in North America via vegetative propagules and/or fragments as populations are only male or have no visible gametangia (Alix, Scribailo, & Weliczko, 2017). Otto and Marks (1996) predicted asexual reproduction should be favored in haplontic life cycles, such as found in the charophytes.

Finally, when meiosis and fertilization are temporally, and often spatially, separated, somatic development occurs in both the haploid and diploid stages (Figure 1c). Bell (1994) called this type of life cycle the Hofmeister–Strasburger alternation of generations. He viewed these life cycles as two distinct vegetative cycles (haploid and diploid) separated by a sexual cycle (meiosis and fertilization). The haploid gametophyte stage produces haploid spores that can either differentiate into a new haploid individual or enter the sexual cycle and undergo fertilization producing a diploid individual. The diploid sporophytes produce diploid spores that can reconstitute a new diploid individual or enter the sexual cycle and undergo meiosis to create new haploid individuals. Bell (1994) argued that gametophyte and sporophyte stages should be viewed as vegetative phases which stand in different relationships to the sexual cycle. Fertilization occurs after haploid gametes are produced via mitosis from the haploid gametophytes, whereas meiosis occurs in the diploid sporophytes. Nevertheless, growth occurs in the haploid gametophyte after meiosis and before fertilization, and, in the sporophyte, growth occurs after fertilization and before meiosis.

In angiosperms, conifers, and ginkgos, the gametophytes are few‐celled and always unisexual, and the life cycle is dominated by the diploid sporophytic stage. Angiosperms are “functionally” diplontic because though there is a gametophytic stage, it is few‐celled, does not undergo substantial haploid somatic development, and is dependent on the sporophyte. While different ploidies have been compared in angiosperm invasions (Bowen et al., 2017), diploid and polyploid angiosperms are not free‐living stages of the same sexual life cycle, but are rather wholly separate life cycles (Wood et al., 2009). In other words, what happens in a diploid lineage does not directly impact the polyploid lineage and vice versa. Mosses, on the other hand, have dominant gametophytes, and the sporophytes develop directly on the gametophytes. Thus, the two vegetative parts of these types of plants are completely overlapping due to dependence of the haploid or diploid stage on the other stage for nutrients, and, as such, do not constitute distinct ecological entities.

In contrast, ferns have dominant sporophytes, and the gametophyte stage (the prothallus) is small and is thought to be rarely seen in nature, but a recent study demonstrated that they are ecologically relevant in which sporophytic and gametophytic communities can differ in species composition and phylogenetic structure (Nitta, Meyer, Taputuarai, & Davis, 2017). Neither the fern gametophyte nor sporophyte is dependent on one another as in mosses or seed plants. Similarly, in kelps, the diploid sporophytes are dominant and macroscopic and the free‐living gametophytes are microscopic (Graham & Wilcox, 2000), but are also ecologically relevant (e.g., Robuchon, Couceiro, Peters, Destombe, & Valero, 2014). Many other macroalgae include multicellular, wholly separate, macroscopic adult haploid gametophytes and diploid sporophytes (there are also similar patterns in the fungi, see Billiard et al., 2012). It is the independence of these generations in fern and macroalgal life cycles that have important evolutionary consequences. The gametophytes and sporophytes are components of the same sexual life cycle, connected by meiosis and fertilization, and ecological factors influencing gametophytes directly impact sporophytes and vice versa. The exception may be the red macroalgae. However, while red macroalgae with a *Polysiphonia*‐type life cycle have a third carposporophyte stage (the stage where the zygote is mitotically amplified following fertilization), it is retained on the female gametophyte and is not free‐living (Searles, 1980). Moreover, this stage is genetically identical to the sporophyte stage it will produce; thus, red algal life cycles are biphasic (Engel et al., 1999; Krueger‐Hadfield et al., 2015). For the purposes of assessing mating system dynamics, a red algal haplodiplontic life cycle is not functionally different from brown and green algal haplodiplontic life cycles (Engel et al., 1999; Krueger‐Hadfield et al., 2015). Nevertheless, in any haplodiplontic life cycle in which the gametophytes and sporophytes are free‐living, the spatiotemporal separation of meiosis and fertilization influences ecological and demographic processes, with cascading effects through the whole ecosystem (Krueger‐Hadfield & Hoban, 2016; Thornber, 2006).

While biphasic, haplodiplontic life cycles have received both theoretical attention and empirical attention (e.g., Hughes & Otto, 1999; reviewed in Thornber, 2006), the life cycle literature has overwhelmingly focused on the evolution of diploidy and complex multicellularity (Mable & Otto, 1998; Valero, Richerd, Perrot, & Destombe, 1992). The rather large gap in our understanding for the maintenance of haplodiplontic life cycles (Coelho et al., 2007) limits our understanding of eukaryotic sex and the trade‐offs of sexual versus asexual reproduction (Otto, 2009). In the context of marine invasions, we lack the ability to synthesize and forecast the consequences of invasions by macroalgae as these taxa will experience unique eco‐evolutionary constraints. Reproduction is not necessarily linked to sex, but in haplodiplontic species, the disruption of sex (i.e., the cycling between meiosis and fertilization) necessarily leads to a disruption of the life cycle itself with concomitant effects of the recovery of sexual reproduction (Krueger‐Hadfield et al., 2016). Due to the spatiotemporal separation of meiosis and fertilization and long‐lived haploid stages, selfing can occur in either monoicious or dioicious taxa, selection may purge genetic loads in the haploids, asexuality can lead to the loss of a ploidy stage, and niche differentiation between the two ploidy stages can have drastic demographic effects. I discuss each of these phenomena in turn.

## 4 HAPLODIPLONTIC SELFING

*Consequently, the occurrence of dioicy does not automatically mean that inbreeding is negligible in natural populations.* Valero et al. (2001) *Cahiers de Biologie Marine*, *42*, 53–62

**Figure 2:**
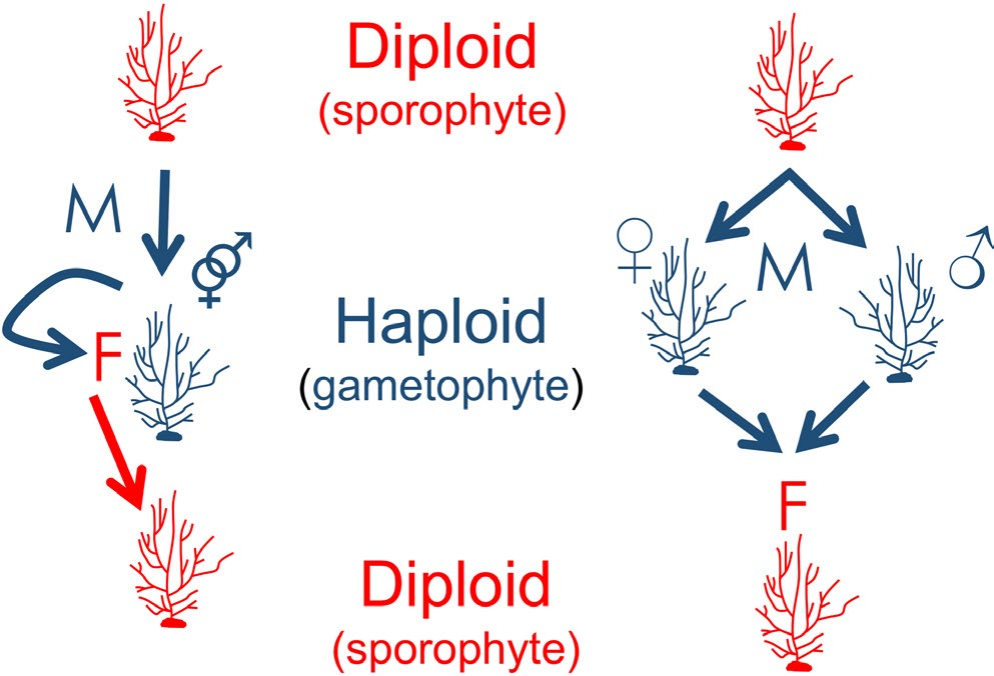
In haplodiplontic life cycles, selfing can occur in monoicious and dioicious species as shown in this schematic. In monoicious species, intergametophytic selfing occurs when uniting gametes are produced by the same haploid thallus. In dioicious species, intergametophytic selfing occurs when uniting gametes are produced by haploid males and females that share the same diploid parent. *M* = meiosis and *F* = fertilization. Diploid stages/processes are shown in red, and haploid stages/processes are shown in blue

The plant literature is replete with eco‐evolutionary studies on the transitions from selfing to dioecy and mixed‐mating systems in natural populations (e.g., Eckert et al., 2010; Winn et al., 2011; Wright, Kalisz, & Slotte, 2012). Self‐fertilization in a typically outcrossing population will erode heterozygosity potentially leading to the expression of deleterious, recessive alleles, ultimately leading to inbreeding depression. In haplodiplontic species, such as mosses, ferns, fungi, or macroalgae, there are two possible ways for selfing to occur (Figure 2), depending on whether or not a species has separate sexes (Beukeboom & Perrin, 2014; Klekowski, 1969; Soltis & Soltis, 1992). Intragametophytic selfing occurs when uniting gametes are produced by the same haploid thallus (i.e., monoicy). This is similar to selfing in hermaphroditic animals and angiosperms, but as gametes are produced by mitosis, and not meiosis, intragametophytic selfing results in instantaneous homozygosity. Dioecy, or separate sexes, is often used as a proxy for an outcrossed mating system, as the evolution of separate sexes was likely driven by selection for outcrossing (Bell, 1997). In haplodiplontic species, intergametophytic selfing involves cross‐fertilization, but between *full‐sib* haploid males and females that share the same diploid parent (Klekowski, 1969). This type of mating is wholly analogous to selfing in animals and angiosperms, and has similar impacts on gene flow and genetic diversity, despite involving cross‐fertilization between two separate, haploid individuals. Thus, the occurrence of dioicy (sex is determined in the haploid stage, Beukeboom & Perrin, 2014) in haplodiplontic species does not automatically result in negligible inbreeding in natural populations (Valero et al., 2001). Indeed, dispersal distance is often very restricted in macroalgae, leading to strong population structuring (Billot, Engel, Rousvoal, Kloareg, & Valero, 2003; Engel et al., 2004; Krueger‐Hadfield, Roze, et al., 2013; Robuchon, Le Gall, Mauger, & Valero, 2014) and likely governing mating unions (Billard, Serrão, Pearson, Destombe, & Valero, 2010; Engel et al., 1999; Krueger‐Hadfield et al., 2015; Maggs et al., 2011). For example, restricted dispersal led to genetic differentiation between low and high intertidal populations in *Chondrus crispus* with only a few meters in tidal height and <50 m in horizontal topographical distance (Krueger‐Hadfield, Roze, et al., 2013).

Understanding the macroalgal axis of variation from selfing to outcrossing is limited by the few taxa that have been studied with appropriate molecular tools. There is evidence of selfing in *C. crispus* (Krueger‐Hadfield, Collen, Daguin‐Thiébaut, & Valero, 2011; Krueger‐Hadfield et al., 2015; Krueger‐Hadfield, Roze, et al., 2013), *Postelsia palmaeformis* (Barner, Pfister, & Wootton, 2011), and *Fucus spiralis* (hermaphroditic/monoecious and diplontic, Billard et al., 2010), but the mating system seems to be allogamous in *Gracilaria gracilis* (Engel et al., 2004) and in some kelps (Billot et al., 2003; Robuchon, Gall, et al., 2014).

Selfing might be advantageous at different stages of colonization during invasions, whether macroalgae are monoicious or dioicious. During initial settlement, there could be mate limitation (Kalisz et al., 2004), coupled with restricted dispersal distance of most algal propagules (Santelices, 1990). Self‐compatibility, which is evident in many macroalgal species (Krueger‐Hadfield et al., 2015; Raimondi, Reed, Gaylord, & Washburn, 2004), should facilitate establishment. Subsequent spread at range edges on invasion fronts will also be mate limited and originating from an already depauperate genetic pool of the initial colonizers. Finally, longer‐term establishment and spread may be facilitated by selfing with the spread of particular, advantageous genotypes (Lynch, 1984).

The highly invasive, dioicious kelp *Undaria pinnatifida* is self‐compatible, and both natural rocky reefs and marina populations showed high levels of selfing in the non‐native range (Guzinski et al., 2018). In contrast, in non‐native farmed populations, farmers mix male and female gametophytes to establish new crops, thereby reducing levels of selfing and inbreeding (Guzinski et al., 2018). Thus, depending on what populations a researcher sampled (non‐native natural/marina vs. non‐native farmed), a different conclusion on the role of selfing would be made. *Sargassum muticum* is a monoecious, diplontic, self‐compatible species, and selfing likely explains its rapid expansion throughout coastal habitats worldwide (Engelen et al., 2015). Molecular investigations of this species have confirmed high selfing rates, though they appear to be more important in established, non‐native populations (Le Cam et al., 2019). The invasive, haplodiplontic, red macroalga *Grateloupia turuturu* is monoicious (Irvine, 1983), but no studies have investigated mating system dynamics during its worldwide invasion. However, the haploid and diploid stages are macroscopic and distinct entities, unlike kelps. Thus, studies are necessary to determine the role of selfing in the expanding front of this seaweed along European and North American coastlines where it may outcompete native taxa (e.g., Kraemer, Yarish, Kim, Zhang, & Lin, 2017).

The combination of asexual reproduction (see below) and selfing can facilitate the propagation of certain genotypes. In the red macroalga *Agarophyton vermicuophyllum* (formerly *Gracilaria vermiculophylla*, Gurgel, Norris, Schmidt, Le, & Fredericq, 2018), *F*_IS_ values in some native and non‐native populations were significantly positive (Krueger‐Hadfield et al., 2016), suggesting that intergametophytic selfing could be occurring. However, though null alleles were negligible across loci (Kollars et al., 2015), spatial substructuring may have inflated *F*_IS_ in some populations, and the relative contributions of subdivision and the mating system remain to be tested in this species. Nevertheless, in some non‐native populations, vegetative fragmentation may propagate certain diploid genotypes through clonal selection that may then disproportionately contribute to the next generation of haploid genotypes when populations encounter hard substratum and reconstitute the sexual life cycle via founder effects. The role of this phenomenon in nature, but future studies, should explore the connectivity between free‐floating diploid‐dominated populations and haploid–diploid, putatively sexual, fixed populations (Krueger‐Hadfield et al., 2018).

## 5 THE HAPLOID STAGE AND SELECTION

*…haploidy has a “longer‐term” advantage, because it allows a better selective elimination of deleterious alleles.* Coelho et al. (2007) *Gene, 406*, 152–170

Higher rates of selfing at any stage of an invasion or along invasion fronts may result in inbreeding depression. While range edge populations may show less inbreeding depression (Pujol et al., 2009), the prolonged haploid stage in haplodiplontic macroalgae may further facilitate purging of deleterious mutations. The haploid stage is a critical window in which selection can act because no deleterious mutation can be masked (Otto & Goldstein, 1992; Otto & Marks, 1996). In the kelp *P. palmaeformis*, there were few costs to selfing, suggesting purging may have already occurred, and selfing may be a mode of reproductive assurance (Barner et al., 2011). In contrast, in the kelp *Macrocystis pyrifera*, the costs to intergametophytic selfing were high, in which selfed sporophytes had low fitness (Raimondi et al., 2004). However, costs were more pronounced later in the life of the sporophyte, suggesting self‐fertilization occurs frequently in natural populations. The red seaweed *C. crispus* exhibited extremely high levels of intergametophytic selfing (Krueger‐Hadfield et al., 2011, 2015; Krueger‐Hadfield, Roze, et al., 2013), but the impacts of inbreeding depression in these populations are unknown.

Inbreeding depression has not been systematically studied across macroalgal taxa as very few data exist describing mating system dynamics (Engel et al., 2004, 1999; Krueger‐Hadfield et al., 2011, 2016, 2015; Krueger‐Hadfield, Roze, et al., 2013) or formal tests for inbreeding depression. The aforementioned examples are not invasive species, but evidence discussed in the previous section clearly demonstrates selfing occurs in invasive macroalgae. Studies explicitly testing the role of haploid selection are critical in the context of invasions in order to understand whether and how selfing in haplodiplontic species constrains adaptive evolution during invasions, as well as in response to other climate change stressors.

## 6 ASEXUALITY AND THE LOSS OF A PLOIDY STAGE

*Despite the large number of clonal [asexual] species present across a wide variety of taxa and habitats, evolutionary theory and models are mostly based on singular genetic individuals.* Arnaud‐Haond, Duarte, Alberto, and Serrao (2007) *Molecular Ecology*, *16*, 5116–5139

**Figure 3:**
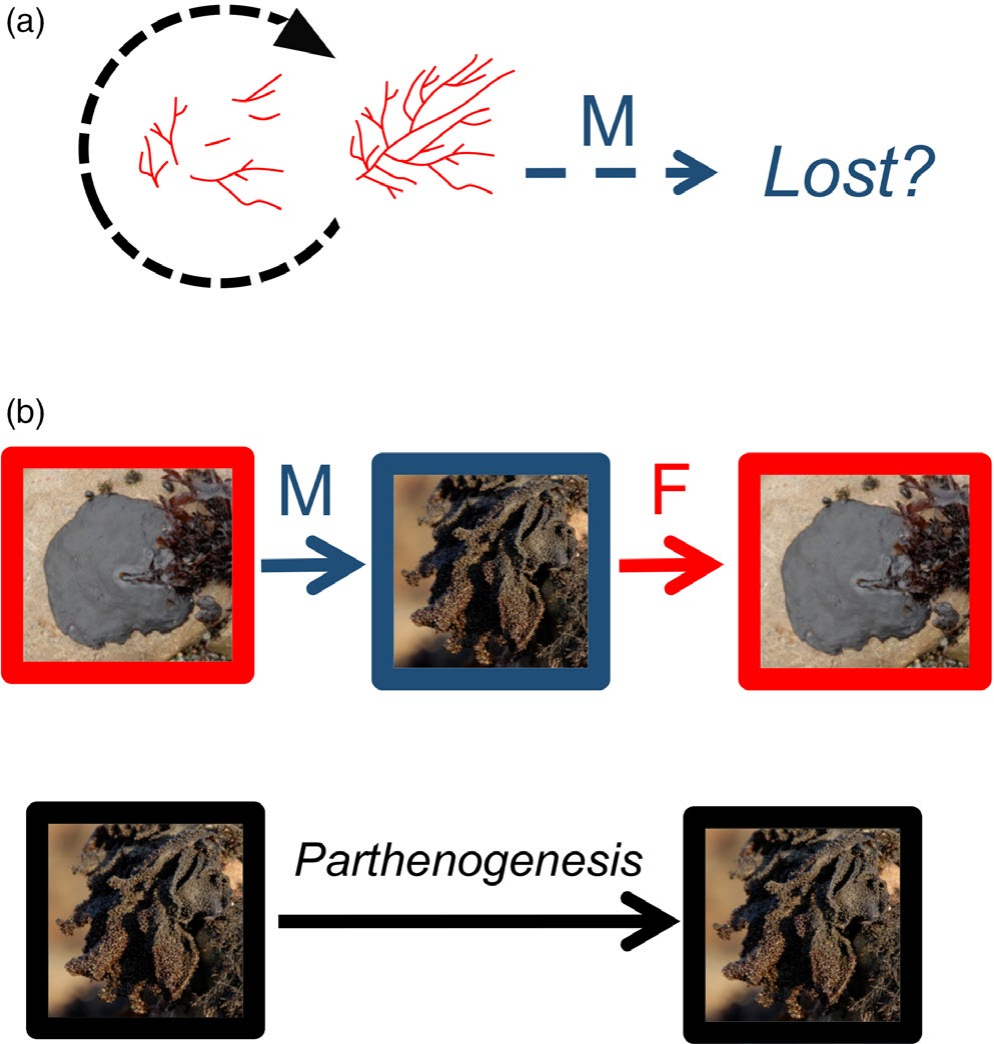
(a) In *Agarophyton vermiculophyllum*, asexual fragmentation in soft‐sediment habitats has resulted in the dominance of diploid sporophytes in many populations. Meiotically produced tetraspores are viable, but are lost in soft‐sediment habitats. (b) In *Mastocarpus* species, crustose diploids undergo meiosis to produce foliose haploids, which in turn undergo fertilization to produce new diploids. The parthenogenetic life cycle involves the recycling of diploid foliose females. *M* = meiosis and *F* = fertilization. Diploid stages/processes are shown in red, haploid stages/processes are shown in blue, and asexual fragmentation and parthenogenesis are shown in black

Asexuality (or clonality in which individuals produce genetically identical progeny through fragmentation, fission, or the production of propagules; de Meeûs, Prugnolle, & Agnew, 2007) should be similarly advantageous as selfing (see above) during colonization and establishment of invading macroalgae by providing reproductive assurance and propagating particular genotypes. However, in a haplodiplontic species, asexuality will result in the dominance of one ploidy stage and potential loss of the other due to the spatiotemporal separation of meiosis and fertilization in haplodiplontic life cycles (Figure 3; Billiard et al., 2012; de Groot et al., 2012; Gabrielson, Brochmann, & Rueness, 2002; Guillemin et al., 2008; Klekowski, 2003; Krueger‐Hadfield et al., 2016; Krueger‐Hadfield, Kübler, & Dudgeon, 2013; Laenen et al., 2015; Maggs, 1988). Unlike diplontic and haplontic life cycles, future “sexual reproductive assurance” will only be maintained if diploids, the stage in which meiosis occurs, are not lost.

The spread of *C. taxifolia* is due in large part to clonal reproduction (Arnaud‐Haond et al., 2013; Meusnier et al., 2004) and has had dramatic ecological consequences (Williams & Smith, 2007). However, in the case of invasive, diplontic *Caulerpa* species, a ploidy stage is not lost and, thus, sexual reproduction is not irrevocably lost. In contrast, in habitats invaded by the red seaweeds *Agarophyton vermiculophyllum* (Krueger‐Hadfield et al., 2016) and *A. chilensis* (Guillemin et al., 2008), the haploid stage has been lost as a consequence of profound ecological (hard to soft substratum) and mating system shifts (sexual to asexual reproduction). However, the presence of reproductive structures on diploid thalli suggests meiosis has not yet been lost. Without sexual recombination to track environmental change, rapid local extinction could befall any of these clonal populations, should unfavorable abiotic or biotic conditions develop, and may have occurred in farmed populations of *A. chilensis* (Leonardi et al., 2006). Apart from one population in Peru (Robitzch, Arakaki, Mauger, Zapata Rojas, & Guillemin, 2019), no haploid‐dominated populations have been found in non‐native habitats. Thus, in these invasive diploid‐dominated habitats, future sexual cycling can be recovered should ecological conditions permit (i.e., hard substratum). In *A. vermiculophyllum*, new surveys have uncovered many more “sexual” populations fixed to hard substratum in which reproductive haploid and diploid individuals are common (Krueger‐Hadfield et al., 2018). Molecular investigations are necessary to determine whether haploid and diploid stages are regularly connected through sex (see as examples in other taxa: Engel et al., 2004; Krueger‐Hadfield, Roze, et al., 2013). Moreover, more detailed within‐site surveys have uncovered small pockets of fixed subpopulations in otherwise diploid‐dominated, free‐floating populations of *A. vermiculophyllum* (S. A. Krueger‐Hadfield, unpublished data). It is unclear whether these are newly settled recruits with an increase in hard substratum as anthropogenic disturbance increases in some regions, or whether these fixed thalli were overlooked in previous surveys. In short, in many free‐floating *A. vermiculophyllum* populations, if hard substratum is provided, recruits will appear (see also Lees et al., 2018).

In the case of *Mastocarpus*, a heteromorphic, red algal species complex in the North Pacific, asexuality results in the loss of the diploid stage and a novel ploidy and morphological combination (Dudgeon, Kübler, West, Kamiya, & Krueger‐Hadfield, 2017). Asexual females have the morphology of the haploid gametophyte, but the ploidy level of the diploid sporophyte (Dudgeon et al., 2017; Maggs, 1988). The loss of the diploid stage enables asexual lineages to expand along latitudinal and tidal gradients that correspond to changes in ecological gradients at the macro‐ and microscale, respectfully (Fierst, Kübler, & Dudgeon, 2010; Krueger‐Hadfield, Kübler, et al., 2013). However, asexual females cannot revert back to sexual reproduction, so they are forever shunted off to the asexual cycle (reviewed in Dudgeon et al., 2017). One of these *Mastocarpus* species is present near Concépcion, Chile, and is considered to be non‐native (Orostica, Otaiza, & Neill, 2012). The typical sexual alternation of diploids and haploids as well as asexual females are found at sites in Chile, but males are rare (Avila & Alveal, 1987). Asexual females producing mixed progeny or reverting to the sexual haplodiplontic life cycles are very rare in these Pacific *Mastocarpus* species (Dudgeon et al., 2017; Guiry & West, 1983; Maggs, 1988; Polanshek & West, 1977), but did occur in *Mastocarpus stellatus* in the North Atlantic, suggesting it may be possible in species in the North Pacific. Moreover, there may be monoicious haploids (Lindstrom, Hughey, & Martone, 2011; Maggs, 1988) that can undergo self‐fertilization, but the life cycles of variants in this Pacific *Mastocarpus* species complex are unknown. Thus, monoicious haploids may have facilitated the invasion of *Mastocarpus* sp. with subsequent spread through a combination of monoicy, asexual reproduction, or both, but molecular investigations are necessary in order to delineate the invasion history of this species in Chile.

## 7 NICHE DIFFERENTIATION OF HAPLODIPLONTIC LIFE CYCLE STAGES

*In order for biphasic [haplodiplontic] life cycles to be favored, it does not matter what the particular differences between the two ploidy phases are, only that they are sufficiently different to exploit an environment more efficiently together than either could do alone.* Hughes and Otto, (1999) *The American Naturalist*, *154,* 306–320

The maintenance of haplodiplontic life cycles is hypothesized to be driven by ecological niche differentiation (Hughes & Otto, 1999). In heteromorphic life cycles, such as the *Mastocarpus* example from above, morphologically distinct haploids and diploids occupy spatiotemporally heterogeneous environments and are better able to exploit these unpredictable conditions together (e.g., bet‐hedging, Lubchenco & Cubit, 1980). Even though isomorphic life cycles have been considered ecologically identical (Klinger, 1993; Valero et al., 1992), studies have described cryptic survival and resource differences between ploidies that may be as ecologically relevant as found in heteromorphic life cycles (Destombe, Godin, Lefèvre, Dehorter, & Vernet, 1992; Engel & Destombe, 2002; Guillemin, Sepúlveda, Correa, & Destombe, 2013; Krueger‐Hadfield, 2011; Lees et al., 2018; Thornber & Gaines, 2004). Indeed, Hughes and Otto (1999) suggested very subtle phenotypic differences at any life stage are sufficient to stabilize haplodiplontic life cycles over evolutionary timescales. Yet, these phenotypic studies are taxonomically restricted (Thornber, 2006) and not as numerous as is necessary to critically assess the consequences of changing selective pressures that may render haplodiplontic species vulnerable to selection against the “weakest link” (Istock, 1967). Moreover, haploids and diploids may differ in their plasticity, but too few studies have assessed these patterns at the genotype level from which meaningful syntheses could be drawn.

This is especially true in the context of marine invasions, where only a handful of studies have empirically tested the predictions of Hughes and Otto (1999). In the case of *A. vermiculophyllum*, asexual reproduction contributed to the dominance of diploid stage, but differences in palatability and thallus integrity likely led to the loss of the haploid thalli in invasive populations over successive clonal generations (Lees et al., 2018). Even though the diploid stage is where meiosis occurs, if haploids are unable to exist in some of these invasive populations where *A. vermiculophyllum* is now found, future sexual reproduction may not be possible, and the life cycle will remain interrupted and uncoupled. Compounded by the lack of genomic information for haplodiplontic species (Coelho et al., 2013; Collen et al., 2014), general syntheses are impossible, restricting our ability to forecast the consequences of climate change (Mable & Otto, 1998; Valero et al., 1992), and evolution during invasion in particular.

## 8 CONCLUSIONS

*Additional studies of the natural history and genetics of algae are sorely needed to further our understanding of the evolution of life cycles and to test theories …* Otto and Marks (1996) *Biological Journal of the Linnean Society, 57,* 197–218

Few studies integrate both ecological and evolutionary processes in understanding responses to contemporary climate change (Anderson, Panetta, & Mitchell‐Olds, 2012; Rey et al., 2012), and specifically biological invasions. This is in part because substantial amounts of information are necessary in order to distinguish between different eco‐evolutionary scenarios that facilitate invasions (Hufbauer et al., 2012), including (a) phenotypic data for native and non‐native populations, (b) genetic data for documenting the invasion history, including mating system variation, and (c) biotic and abiotic environmental data to assess selection pressures acting across the extant range (Rey et al., 2012). There are many unanswered questions about the eco‐evolutionary dynamics of invasions (e.g., the independent and interactive effects of plasticity, adaptation, and population demography), and these are the topic of ongoing discussion and exploration. However, an essential component of this discussion must be a more nuanced examination of the effect of mating system on invasion success.

**Figure 4:**
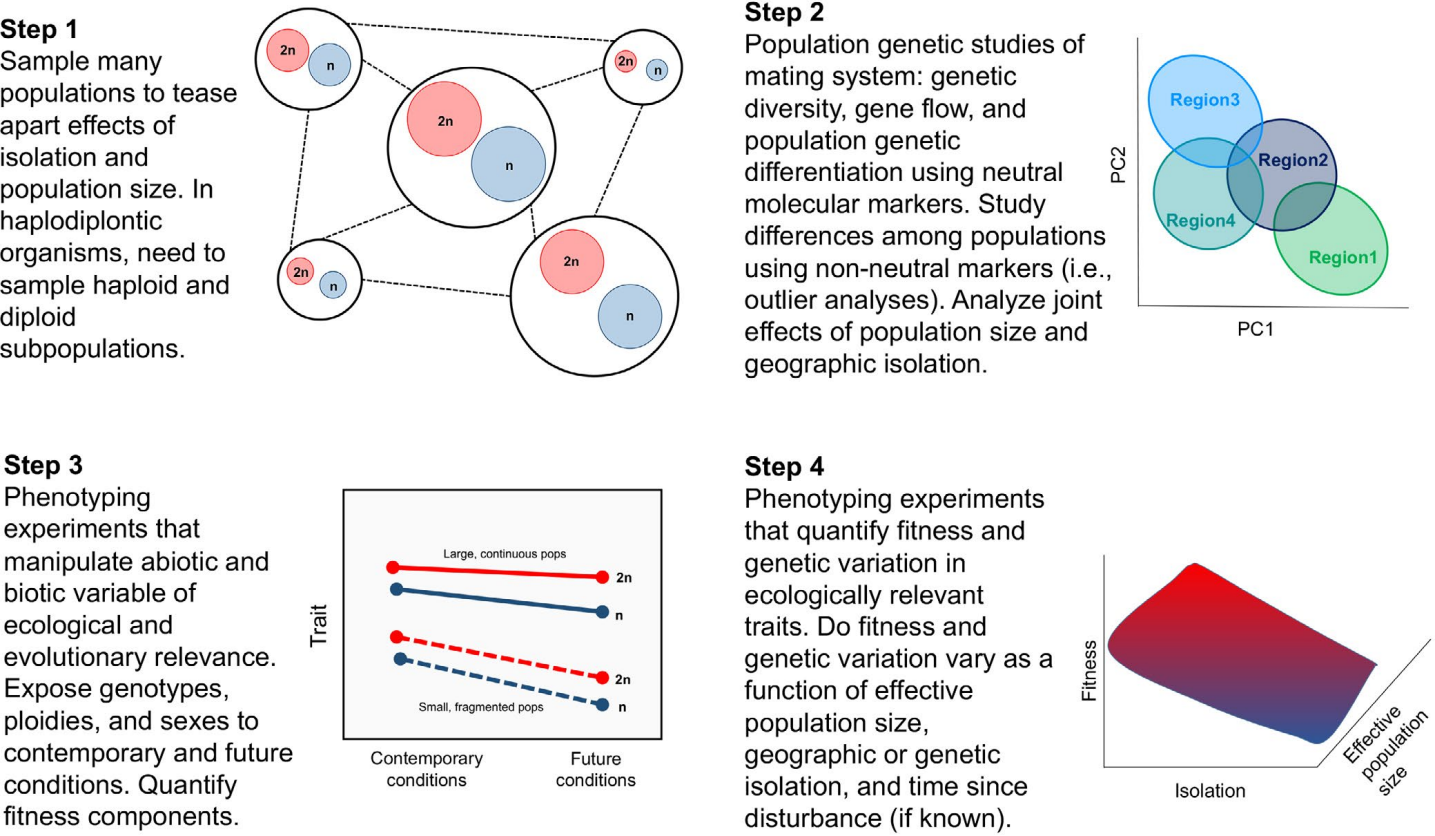
Schematic of steps with which to develop necessary information with which to test the role of evolution in marine invasions, especially in haplodiplontic macroalgae, adapted from Anderson et al. (2012). In Step 1, studies need to sample populations across a range of demographic and ecological variables. In haplodiplontic species, this requires exhaustive sampling to capture genetic parameters in both the haploid and diploid stages (Krueger‐Hadfield & Hoban, 2016). Step 2 will assess population genetic and genomic characteristics of populations, with particular emphasis on the mating system and geographic structure (shown as a discriminant principal components analysis of four regions). Step 3 will employ common garden experiments to assess phenotypic differentiation among populations and within life cycle stages, including diploids and haploid male and females. Finally, Step 4 will include experiments that expose genotypes to a range of abiotic and biotic stressors and assess fitness components

Anderson et al. (2012) provided a predictive framework to test the evolutionary responses to climate change. Adaptive potential relies on the amount of extant genetic variation and the heritability of ecologically relevant traits. We must use this framework in order to explicitly address these phenomena in haplodiplontic species, and integrate the explicit characterization of the intersection between mating system and life cycle dynamics (Figure 4). Since we lack fundamental information about mating system variation, for the purposes of this perspective, and marine invasions in general, we need to first generate critical data for steps 1 (spatially explicit sampling of haplodiplontic populations), 2 (genetic analyses of life cycle stages, mating system variation, and genetic structure), and 3 (phenotyping experiments across ploidies, sexes, and populations) before we can truly test the adaptive potential of these species in response to climate change (i.e., step 4). While haplodiplontic species have unique eco‐evolutionary patterns (i.e., possibility of selfing even if there are separate sexes), generalized patterns should be followed in the study of marine invasions in order to describe the evolution of mating systems and life cycles in order to understand the role that evolution plays in potential migration, and then subsequent adaptation, plasticity, and evolutionary potential (Pannell, 2015). Widespread invaders, such as *S. muticum* (Le Cam et al., 2019)*, C. taxifolia* (Meusnier et al., 2004), *U. pinnatifida* (Guzinski et al., 2018)*, Agarophyton vermiculophyllum* (Krueger‐Hadfield et al., 2016), and *G. turuturu*, would enable us to test the relative contributions of life cycle and mating system variation to invasion success in marine ecosystems. This is by no means an exhaustive list of possible models, but rather macroalgae that differ in monoe‐/monoicy and dioe‐/dioicy and life cycle characteristics allowing us to determine the extent to which invasion patterns are global across taxa.

## CONFLICT OF INTEREST

None declared.

## Data Availability

I will not be archiving data because this manuscript does not have associated data.
